# Targeted Deletion of the ERK5 MAP Kinase Impairs Neuronal Differentiation, Migration, and Survival during Adult Neurogenesis in the Olfactory Bulb

**DOI:** 10.1371/journal.pone.0061948

**Published:** 2013-04-22

**Authors:** Tan Li, Yung-Wei Pan, Wenbin Wang, Glen Abel, Junhui Zou, Lihong Xu, Daniel R. Storm, Zhengui Xia

**Affiliations:** 1 Toxicology Program in the Department of Environmental and Occupational Health Sciences, University of Washington, Seattle, Washington, United States of America; 2 Department of Biochemistry and Genetics, Zhejiang University, Hangzhou, Zhejiang, China; 3 Graduate Program in Molecular and Cellular Biology, University of Washington, Seattle, Washington, United States of America; 4 Department of Pharmacology, University of Washington, Seattle, Washington, United States of America; Université Lyon, France

## Abstract

Recent studies have led to the exciting idea that adult-born neurons in the olfactory bulb (OB) may be critical for complex forms of olfactory behavior in mice. However, signaling mechanisms regulating adult OB neurogenesis are not well defined. We recently reported that extracellular signal-regulated kinase (ERK) 5, a MAP kinase, is specifically expressed in neurogenic regions within the adult brain. This pattern of expression suggests a role for ERK5 in the regulation of adult OB neurogenesis. Indeed, we previously reported that conditional deletion of *erk5* in adult neurogenic regions impairs several forms of olfactory behavior in mice. Thus, it is important to understand how ERK5 regulates adult neurogenesis in the OB. Here we present evidence that shRNA suppression of ERK5 in adult neural stem/progenitor cells isolated from the subventricular zone (SVZ) reduces neurogenesis in culture. By contrast, ectopic activation of endogenous ERK5 signaling via expression of constitutive active MEK5, an upstream activating kinase for ERK5, stimulates neurogenesis. Furthermore, inducible and conditional deletion of *erk5* specifically in the neurogenic regions of the adult mouse brain interferes with cell cycle exit of neuroblasts, impairs chain migration along the rostral migratory stream and radial migration into the OB. It also inhibits neuronal differentiation and survival. These data suggest that ERK5 regulates multiple aspects of adult OB neurogenesis and provide new insights concerning signaling mechanisms governing adult neurogenesis in the SVZ-OB axis.

## Introduction

The ability of humans and other vertebrates to detect and distinguish between thousands of different odorants is critical for nutrition and survival. Normal ongoing adult neurogenesis occurs in two principle regions of the adult mammalian brain including the subventricular zone (SVZ) along the lateral ventricles (LV) [1], [2], [3], [4]. Newly generated neuronal precursors in the SVZ migrate along the rostral migratory stream (RMS) to the core of the olfactory bulb (OB) where they differentiate into inhibitory periglomerular cells (about 5%) and granule cells (about 95%) [5], [6], [7], [8]. Recent studies have led to the interesting finding that adult neurogenesis in the OB may influence several complex forms of olfactory behavior in mice [5], [9], [10], [11], [12], [13], [14], [15]. In addition, adult neurogenesis in the OB is a model system to study stem cell biology, regenerative and reparative medicine, and plasticity of the adult nervous system. Thus, it is critical to elucidate signaling mechanisms regulating adult SVZ-OB neurogenesis.

The generation of adult-born OB neurons is a multistep process that includes proliferation, migration, neuronal differentiation and maturation as well as survival. In the adult SVZ, neural precursors (type B and C cells) commit to a neuronal fate and become neuroblasts (type A cells), which can either continue proliferating or exit the cell cycle and become immature neurons. Neuroblasts migrate tangentially along the RMS to the core of the OB and become immature neurons [5], [6], [7], [8]. Upon arrival at the OB, immature neurons switch to radial migration into the granule cell layer (GCL) and periglomerular layer, and undergo terminal differentiation along the way into mature neurons [16]. Many adult-born neurons die by apoptosis in an activity-dependent manner during dendritic branching and synaptogenesis at the OB [16], [17], [18], [19].

ERK5 is a member of the mitogen-activated protein (MAP) kinase family that includes ERK1/2, JNK and p38 [20], [21]. Biochemically, ERK5 is specifically activated by MEK5, a MAP kinase kinase [20], [21]. We recently discovered that although ERK5 expression in the adult brain is extremely low, it is prominently expressed along the neurogenic SVZ-RMS-core of the OB axis [15]. Furthermore, deletion of *erk5* in adult neurogenic regions reduced the percentage of adult-born mature neurons in the OB and impaired several forms of olfactory behavior [15]. These data strongly suggest an important role for ERK5 signaling in regulating adult neurogenesis in the SVZ-OB. In this study, we aim to elucidate mechanisms by which ERK5 regulates adult neurogenesis in the SVZ-OB. Specifically, using RNAi technology and an inducible and conditional knockout (icKO) line of ERK5 transgenic mice, which allows us to delete the *erk5* gene specifically in neurogenic regions of the adult brain, we investigated whether ERK5 deletion affects cell cycle regulation, migration, neuronal differentiation, maturation, and/or survival of adult-born neurons. We report evidence that *erk5* deletion interferes with each and all of these processes, suggesting that ERK5 may play a critical role in multiple aspects of adult neurogenesis in the SVZ-OB axis.

## Materials and Methods

### Ethics Statement

All animals used in this study were approved by the University of Washington Institutional Animal Care and Use Committee. Experimental conditions and procedures were performed with direct approval under protocol 3041–04.

### Animals

The generation of Nestin-CreER™ [22], ERK5^loxP/loxP^ [23], *Gt(ROSA)26Sor-YFP* (R26-YFP) [24], Nestin-CreER™/ERK5^loxP/loxP^ [25], and Nestin-CreER™/R26-YFP^loxP/loxP^ [26] mice have been described. Nestin-CreER™/ERK5^loxP/loxP^ mice and R26-YFP^loxP/loxP^ mice were crossed to yield Nestin-CreER™/ERK5^loxP/loxP^/R26-YFP^loxP/loxP^ mice. All animal experiments were performed with identically treated and handled littermate controls, in mixed SV129/C57BL/6 background. Animals were housed under standard conditions (12 h light/dark cycle) with food and water provided *ad libitum*.

### Reagents

The following primary antibodies and dilutions were used in immunohistochemistry: rat monoclonal anti-BrdU (1∶500 AbD Serotec); mouse antibody against GFAP (1∶1000, Millipore), PCNA (1∶500, Novocastra), SOX2 (1∶200, R&D system), PSA-NCAM (1∶200, Developmental Studies Hybridoma Bank), NeuN (1∶500, Millipore); rabbit antibody against Ki67 (1∶200, Novocastra), active caspase-3 (1∶200, Cell Signaling Technology), GFP (1∶500, Invitrogen); goat anti-DCX (1∶200, Santa Cruz Biotechnology). Rabbit polyclonal ERK5 antibody (1∶500) was generated previously and affinity purified using recombinant MBP-ERK5 protein. The primary antibodies used in immunocytochemistry were: mouse antibody against PCNA (1∶500, Millipore), SOX2 (1∶500, R&D system), β-III tubulin (1∶1000, Promega), GFAP (1∶500, Millipore), O4 (1∶50, Sigma); rabbit anti-GFP (1∶1000, Invitrogen).

### Adult Neural Precursor Cell Culture and in vitro Analysis

Primary cell cultures were prepared as described [27]. Briefly, tissue samples from 8-week-old C57BL/6 mice containing the SVZ were micro-dissected and enzymatically digested with 0.125% trypsin-EDTA for 7 min at 37°C followed by incubation with an equal volume of 0.014% trypsin inhibitor (Invitrogen). Tissue samples were then spun down and resuspended in serum-free culture media consisting of DMEM/F12 (Invitrogen), 1× N2 supplement (Invitrogen), 1× B27 supplement without retinoic acid (Invitrogen), 100 U/mL penicillin/streptomycin (Invitrogen), 2 mM L-glutamine (Invitrogen), 2 µg/mL heparin (Sigma), 20 ng/mL EGF (EMD Chemicals), and 10 ng/mL bFGF (Millipore). Tissue was mechanically triturated and filtered through a 40 µm cell sieve, plated in petri dishes, and cultured for 7–14 d until primary neurospheres formed. EGF and bFGF were replenished every 3 d during this period. Spheres collected from secondary passage were dissociated and plated as a monolayer culture on fibronectin and poly-L-ornithine (BD Biosciences)-coated culture plates or aclar coverslips (Electron Microscopy Sciences) for experiments.

For *in vitro* knockdown or activation of ERK5, cells were infected with shERK5 or constitutively active MEK5 (caMEK5) retrovirus, respectively. A non-specific retrovirus (shNS) was used as a control for shERK5, while the pXIE empty vector was used as a control for caMEK5 as described [26]. Briefly, cells were plated at a density of 50,000 cells per well on coated coverslips in 48-well plates, infected with retroviruses in the presence of 8 µg/mL of protamine sulfate for 24 h. shERK5 and control virus-infected cells were then incubated in EGF- and bFGF-free medium for 5 d to allow spontaneous differentiation in order to determine if knockdown of ERK5 affects spontaneous neuronal differentiation of SVZ-derived neural precursors. However, to detect if activation of ERK5 is sufficient to stimulate neuronal differentiation, caMEK5 and control retroviruses-infected cells were then incubated for 5 d in regular growth medium containing mitogenic growth factors EGF and bFGF.

### Immunocytochemistry

Cells were fixed with 4% paraformaldehyde and 4% sucrose in PBS at room temperature for 30 min. Fixed cells were washed 3×5 min in PBS, 5 min in 1% SDS for permeabilization, and 3×5 min in PBS. Cells were blocked with 5% bovine serum albumin (BSA) dissolved in PBST (PBS with 0.1% Triton X-100) for 2 h, followed by incubation with primary antibodies overnight at 4°C. Cells were then washed 3×10 min in PBST, followed by incubation with secondary antibodies at 1∶5,000 dilution (Alexa-Fluor-488) or 1∶2,000 dilution (Alexa-Fluor-594) for 2 h in blocking buffer. Cells were washed 3×10 min in PBST followed by a 10 min incubation in Hoechst 33342 (Invitrogen) for nuclei visualization and a final wash of 10 min in PBST prior to mounting onto slides using anti-fade Aqua Poly/Mount solution (Polysciences).

### Tamoxifen and Bromodeoxyuridine (BrdU) Administration

Tamoxifen (Sigma) was made freshly and dissolved in 2% glacial acetic acid in corn oil solution (Sigma). Pre-warmed tamoxifen was administered orally to 10–12 week-old mice daily (200 mg/kg) for 7 d (for 20 h BrdU study, and for Nestin-CreER™/R26-YFP^loxP/loxP^ and Nestin-CreER™/ERK5^loxP/loxP^/R26-YFP^loxP/loxP^ mice), or once a day for 4 days repeated for 3 rounds, with 2-week recovery between each round of tamoxifen dosing (for all other studies). Both tamoxifen treatment protocols have been shown to successfully knock down ERK5 expression by at least 65% [25]. BrdU (Sigma) was dissolved in 0.007% NaOH in sterile saline and administered at 100 mg/kg by intra-peritoneal injection once (for examinations at 2 h and 20 h post-BrdU), or 5 times (every 2 h for 10 h) in 1 day (for examinations at 7 d, 14 d, 28 d, and 42 d post-BrdU).

### Immunohistochemistry

Mice were perfused intracardially with ice-cold solutions of 20 mL of PBS, followed by 20 mL of 4% PFA in PBS. Brains were harvested and post-fixed with 4% PFA/PBS overnight at 4°C, followed by 30% (w/v) sucrose in PBS solution at 4°C until brains sunk. Immediately after sucrose embedding, brains were frozen at −80°C. Immunohistochemistry (IHC) was performed on 20 µm-thick coronal sections using a free-floating antibody staining method, or on 14 µm-thick sagittal sections mounted on Superfrost Plus slides (VWR). Briefly, brain sections were washed 4×10 min with PBS, followed by 3×10 min with PBST (PBS plus 0.25% Triton X-100). Brain slices were then incubated in blocking buffer (PBST plus 0.1% BSA and 10% normal serum of the same species from which the corresponding secondary antibodies were derived) for 2 h. For the visualization of BrdU, HCl treatment was performed before the PBST washes and blocking: brain sections were sequentially incubated in water for 5 min, ice-cold 1 N HCl for 10 min, and 2N HCl for 30 min at 37°C; and then neutralized by rinsing 2×20 min in 0.5 M borate buffer, pH 8.5. Brain sections were incubated with primary antibodies for 48 h at 4°C, washed in PBST 4×10 min, and incubated with secondary antibodies conjugated with Alexa-Fluor dyes (1∶500 dilution; Invitrogen) in blocking buffer for 2 h. Brain sections were then washed 4×10 min in PBST, incubated with 2.5 µg/ml Hoechst 33342 (Invitrogen) in PBST for 30 min, and washed 3×5 min with PBST. Unless otherwise stated, all IHC procedures were carried out at room temperature. Free-floating brain sections were then mounted on gelatin-coated Superfrost Plus slides (VWR) with anti-fade Aqua Poly/Mount. Sagittal sections already on Superfrost Plus slides were also mounted with anti-fade Aqua Poly/Mount.

### TUNEL Assay

This was carried out per manufacturer’s (Promega) instruction following immunohistochemistry.

### Confocal Imaging and Analysis

All images were acquired with an Olympus Fluoview-1000 laser scanning confocal microscope with a numerical aperture 0.75, 20× lens or a numerical aperture 1.3, 40× oil immersion lens. Optical Z-sections (0.5–1 µm) were collected and processed by ImageJ (NIH). Images were uniformly adjusted for color, brightness, and contrast with Adobe Photoshop CS6 (Adobe Systems, Inc).

### Quantification of Immunostained Cells

For Immunocytochemistry, at least 200 GFP^+^ cells from each sample were randomly selected and quantified for the co-labeling with individual makers. For immunohistochemistry, every fourth sagittal serial section or every sixth coronal serial section was used for quantification. The density of BrdU^+^ cells, BrdU and NeuN co-labeled cells, and YFP^+^ cells in the OB were quantified using the Optical Fractionator Probe of Stereology Investigator Software (MBF Bioscience), a well-established method to quantify cell numbers without bias [28], [29], [30]. Using a Zeiss upright fluorescence microscope, the region of interest, the GCL, was outlined at 5× magnification based on NeuN or Hoechst staining. The entire area of the GCL was then divided by 120×120 µm^2^ grids. For each stained section, the grids were superimposed on the staining image at a random starting point. A 50×50 µm^2^ counting frame was automatically set up at the upper left corner within each grid. The images were enlarged to higher magnification (40× objective) to allow clear identification of individually stained cells, and the number of BrdU^+^, NeuN^+^/BrdU^+^, or YFP^+^ cells within the GCL boundary and inside of each counting frame were counted. Therefore, all BrdU-labeled, BrdU/NeuN co-labeled, or YFP-labeled cells within the entire GCL were systematically sampled without bias, from all spatial distributions, and all the areas along the external-internal axis of the GCL.

The density of TUNEL/YFP co-labeled cells was calculated by dividing the region’s total labeled-cell count by the volume of that region, as measured by Stereology Investigator Software. The quantification of BrdU^+^ cells in the SVZ and RMS was counted throughout the whole region. The co-labeling of BrdU with Ki67 and DCX was randomly sampled throughout the SVZ and RMS. At least 500 cells were sampled for each data point. The intensity of ERK5 and Ki67 staining was quantified by ImageJ. The average dendritic length and branching number were measured by Simple Neurite Tracer (NIH), with a 3D viewer, from at least 100 cells sampled from each animal.

### Statistical Analysis

All data were obtained from n≥3 mice and expressed as mean ± standard error of the means (s.e.m.). Means between control and icKO mice, or between control and shERK5/caMEK5 infected cells, were analyzed by unpaired two-tail Student *t*-test. n.s. not significant; *, p<0.05; **, p<0.01; ***, p<0.001.

## Results

### ERK5 Protein Expression in the SVZ and RMS

**Figure 1:**
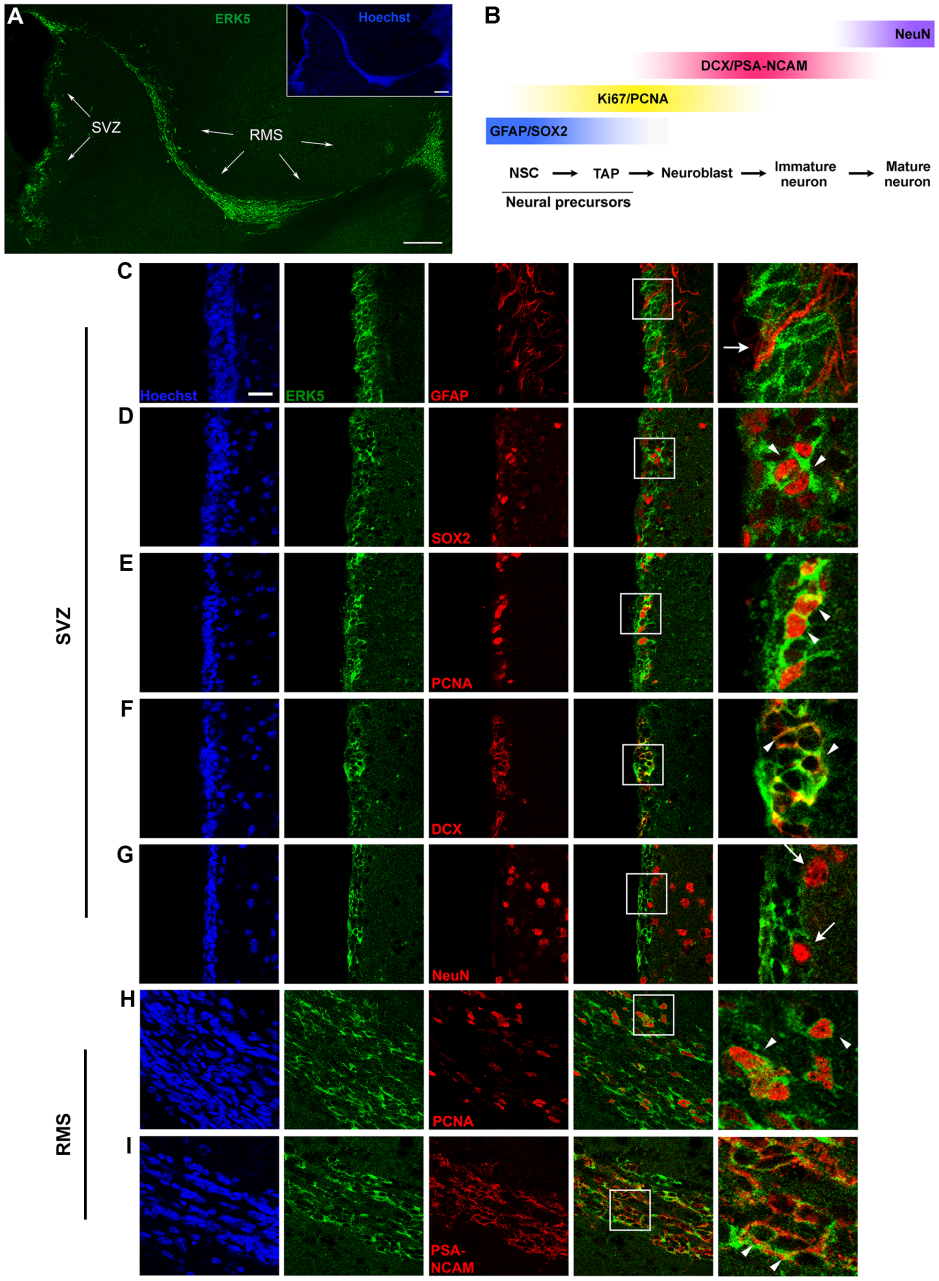
Characterization of ERK5 expression in the SVZ-RMS axis. (A) ERK5 (green) was expressed throughout the SVZ-RMS axis in sagittal sections. Nuclei (blue) were visualized by Hoechst staining. Scale bar represents 250 µm. (B) Schematic diagram of adult OB neurogenesis and markers used in *in vivo* studies. NSC, neural stem cell; TAP, transit-amplifying progenitor. (C-I) Representative confocal images of coronal (SVZ) and sagittal (RMS) brain sections, co-immunostained for ERK5 (green) and GFAP (C), SOX2 (D), PCNA (E), doublecortin (DCX) (F), or NeuN (G) in the SVZ, and PCNA (H), or PSA-NCAM (I) in the RMS. The panels in the far right column represent the enlarged boxed areas respective to their left panels. Scale bar in (C) represents 25 µm and applies to all panels in (C-I), except the right column. Arrowheads point to co-labeled cells, while arrows point to cells that do not express ERK5.

We reported that ERK5 is specifically expressed in the subgranular zone (SGZ) of the dentate gyrus and in the SVZ-RMS of the adult mouse brain [15], [25],[26],[27]. Here, we used an affinity-purified anti-ERK5 antibody to confirm ERK5 expression throughout the SVZ-RMS axis (Fig. 1A) and to further characterize ERK5 expression in the SVZ and RMS. To identify the specific cell types expressing ERK5, we co-stained ERK5 with GFAP, a quiescent stem cell marker in the SVZ; SOX2, a proliferating stem/precursor cell marker; PCNA, a cell proliferation marker; doublecortin (DCX) or polysialylated-neural cell adhesion molecule (PSA-NCAM), markers for neuroblasts/immature neurons; and NeuN, a mature neuron marker (Fig. 1B). We have previously demonstrated co-staining of ERK5 with Pax6 [27], another commonly used marker for SVZ stem/precursor cells [31]. There were no ERK5 and GFAP co-expressing cells found after careful examination of more than 500 ERK5^+^ cells (Fig. 1C). These data suggest that ERK5 is most likely not expressed in quiescent NSCs in the SVZ.

In the SVZ, ERK5 was expressed in proliferating stem/precursor cells (SOX2^+^) (Fig. 1D), proliferating cells (PCNA^+^) (Fig. 1E), and neuroblasts (DCX^+^) (Fig. 1F). We confirmed that almost all PCNA^+^ cells were also Ki67^+^ (data not shown), indicating that PCNA is a suitable proliferation marker under these experimental conditions. In contrast, among 500 ERK5^+^ cells analyzed, none expressed NeuN (Fig. 1G). This pattern of ERK5 expression in the SVZ is consistent with our previous report which also quantified the percentage of ERK5^+^ cells expressing these markers [27].

ERK5 was also expressed in proliferating cells (PCNA^+^) (Fig. 1H), neuroblasts in the RMS (PSA-NCAM^+^) (Fig. 1I), and immature neurons (PSA-NCAM^+^ or DCX^+^) that have reached the core of the OB (data not shown and [15]). The selective expression of ERK5 in the SVZ-RMS, and more specifically in proliferating stem/precursor cells, neuroblasts, and immature neurons led us to hypothesize that ERK5 may regulate adult neurogenesis in the SVZ-RMS-OB axis.

### ERK5 Promotes Neuronal Differentiation of SVZ-derived Neural Precursors *in vitro*

**Figure 2:**
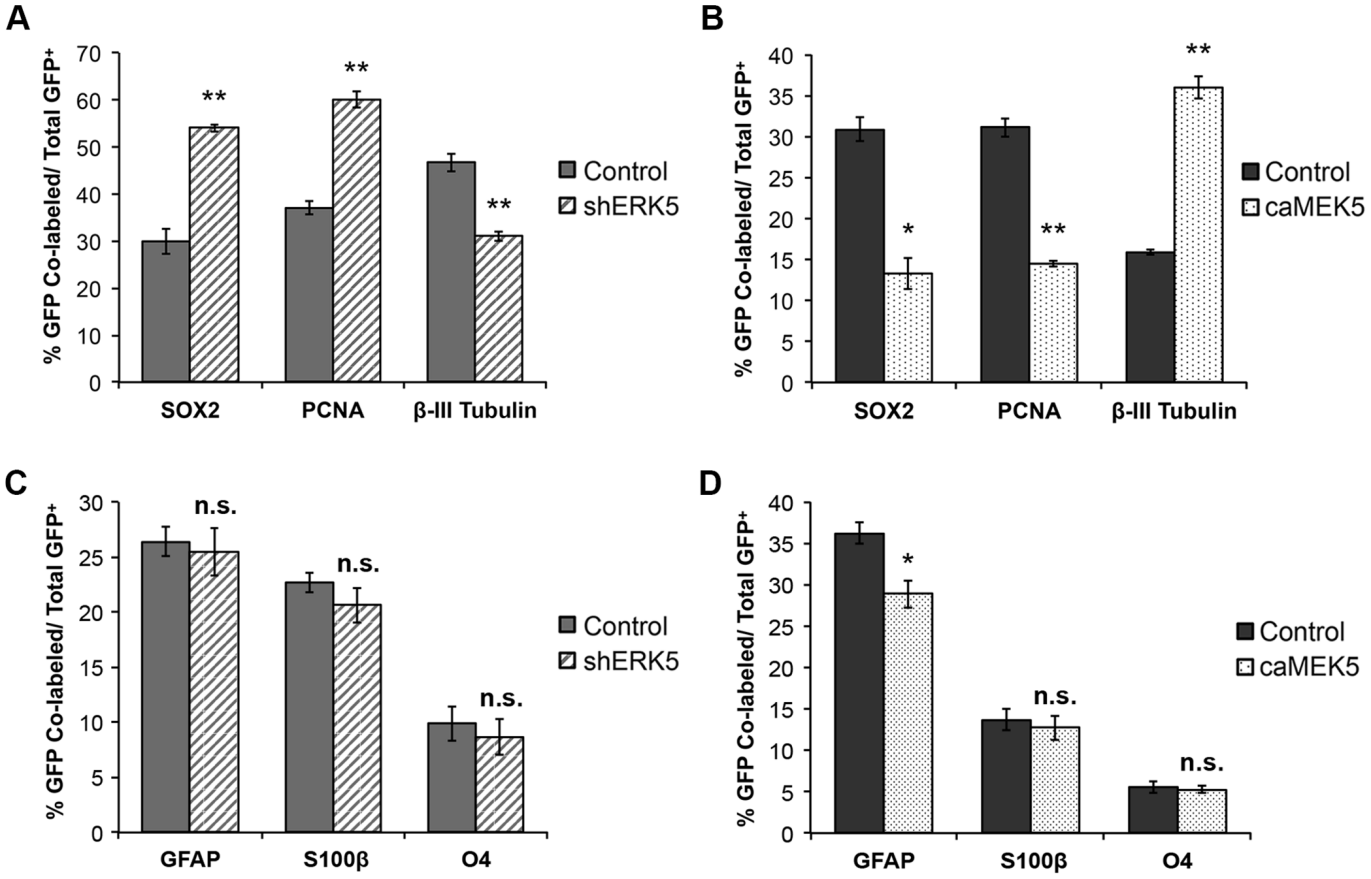
ERK5 signaling regulates neurogenesis of SVZ-derived aNPCs in culture. (A) ERK5 signaling is necessary for promoting spontaneous neurogenesis. aNPCs were infected with non-specific shRNA control retroviral vector (shNS) or shRNA to ERK5 retrovirus (shERK5). Both retroviral vectors encode an eGFP marker protein under a bicistronic promoter [26]. One day after virus infection, cells were incubated in EGF and bFGF-free medium for 5 d to allow spontaneous differentiation. The percentage of GFP^+^ cells that were also SOX2^+^, PCNA^+^, or β-III Tubulin^+^ was quantified. (B) Activation of endogenous ERK5 signaling is sufficient to promote neurogenesis. aNPCs were infected with control retroviral vector expressing eGFP only or expressing caMEK5-IRES-eGFP. One day after virus infection, cells were washed and then placed in fresh regular medium containing mitogenic EGF and bFGF for 5 d. (C) ERK5 knockdown does not affect glial differentiation. GFAP, a marker for astrocytes as well as SVZ stem cells; S100β, a marker for astrocytes; O4, an oligodendrocyte marker. (D) Effect of ERK5 activation on cells expressing GFAP, S100β, and O4. Over 200 virus-infected cells (GFP^+^) from each sample were analyzed and quantified. Data are mean ± SEM from three independent experiments (n = 3). *, p<0.05; **, p<0.01.

We also examined if ERK5 suppression/activation influences glial differentiation. shERK5 did not change the number of GFAP^+^ or O4^+^ cells, markers for astrocytes or oligodendrocytes, respectively, under spontaneous differentiation conditions upon removal of bFGF/EGF from culture media (Fig. 2C). caMEK5 slightly reduced the number of GFAP^+^ cells without affecting O4^+^ cells when aNSCs were cultured in the presence of mitogenic EGF/bFGF (Fig. 2D). Because GFAP is also a marker for SVZ stem/precursor cells when cultured with EGF/bFGF, we performed staining for S100β, a marker for astrocytes that is not expressed in stem/precursor cells [33]. Neither shERK5 nor caMEK5 affected the number of S100β^+^ cells. Thus, ERK5 suppression or activation influences only neuronal differentiation without affecting glial differentiation.

### Inducible and Conditional Deletion of *erk5* in Adult Neurogenic Regions Reduces the Number of Adult-born Neurons in the OB

**Figure 3:**
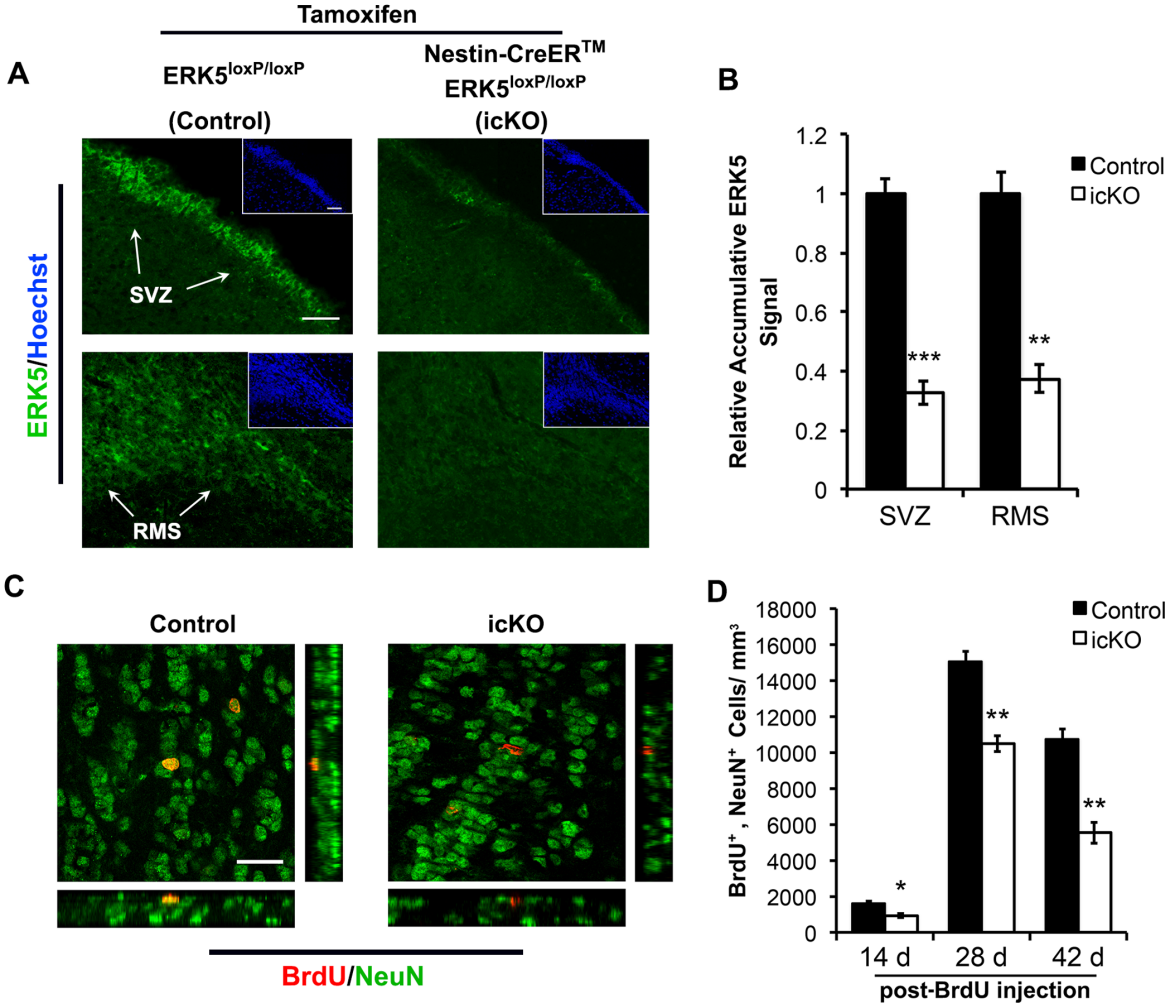
Inducible and conditional knockout (icKO) of ERK5 decreases adult OB neurogenesis. (A) Representative images of ERK5 immunostaining (green) in the SVZ and RMS in control and ERK5 icKO mice. Scale bar represents 50 µm. Insets were Hoechst staining of the sections. (B) Quantification of ERK5 staining intensity in the SVZ and RMS, measured by ImageJ. Data represent accumulative signal relative to control. (C) Representative confocal images of BrdU (red) and NeuN (green) in the OB of control and icKO mice at 28 d post-BrdU injection. Scale bar in (C) represents 20 µm. (D) Quantification of the density of BrdU and NeuN co-labeled cells in the GCL of the OB at 14, 28 and 42 days post-BrdU injection. n  = 4 individual mouse brains and olfactory bulbs per group. *, p<0.05; **, p<0.01; ***, p<0.001.

To determine if ERK5 signaling is required for adult OB neurogenesis *in vivo*, the *erk5* gene was conditionally deleted specifically in the adult neurogenic regions of the mouse brain. This was achieved by tamoxifen administration into adult Nestin-CreER™/ERK5^loxP/loxP^ mice, which inducibly and conditionally deletes the *erk5* gene in Nestin- expressing neural stem/progenitor cells [15], [25]. These mice were designated icKO mice. Identically treated, ERK5^loxP/loxP^ littermates served as controls. Tamoxifen treatment suppressed ERK5 expression in the SVZ and RMS by 65% in icKO mice (Fig. 3A–3B). To label adult-born cells, BrdU was administered 4 d after the last dose of tamoxifen treatment. Adult-born neurons were identified by BrdU and NeuN co-labeling (Fig. 3C). There was a significant reduction of the density of adult-born neurons in the GCL of the OB at 14, 28 or 42 d post-BrdU injection (Fig. 3D). These data suggest that *erk5* deletion attenuates adult neurogenesis of the OB.

### Effect of Conditional Targeting of *erk5* on Cell Proliferation and Cell Cycle Regulation in the SVZ

**Figure 4:**
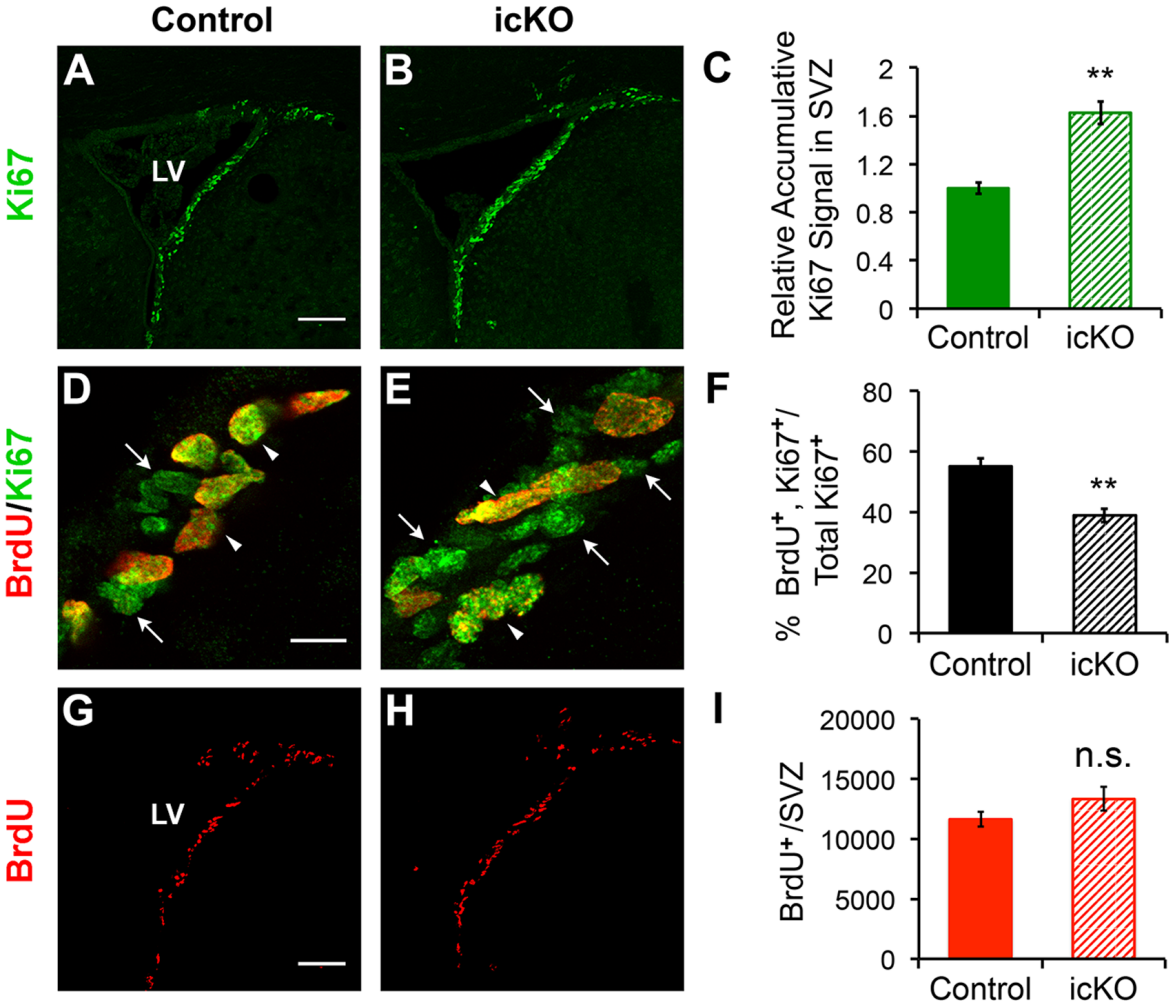
Effect of *erk5* deletion on proliferation in the SVZ. Brain sections were subjected to immunohistochemistry and quantification for Ki67 and BrdU staining. (A-B) Representative images of Ki67 (green) staining in the SVZ of control or ERK5 icKO mice. Scale bar represents 100 µm. (C) Relative intensity of Ki67 staining in the SVZ was measured by ImageJ. (D-E) Representative confocal images of Ki67 (green) and BrdU (red) co-staining in the SVZ at 2 h post-BrdU injection. Arrowheads point to co-labeled cells; arrows point to Ki67^+^ but BrdU^-^ cells. Scale bar represent 10 µm. (F) Percentage of Ki67^+^ cells in the SVZ that are also BrdU^+^. (G-H) Representative images of BrdU (red) staining in the SVZ at 2 h post-BrdU injection. Scale bar represents 100 µm. (I) Total number of BrdU^+^ cells in the SVZ at 2 h after BrdU injection. n  = 4 individual mouse brains and olfactory bulbs per group. n.s. not significant; *, p<0.05; **, p<0.01.

In cancer cells, ERK5 is required for EGF stimulation of cell proliferation; inhibition of ERK5 reduces the number of proliferating cells [34]. To determine if the reduced number of adult-born neurons in the OB of ERK5 icKO mice is due to decreased proliferation, we stained SVZ brain sections with Ki67, a marker for proliferating cells in any stage of the cell cycle. In addition, BrdU was injected into mice 2 h prior to sacrifice to label actively proliferating, S-phase cells. There was an increase in the Ki67 staining intensity in the SVZ of ERK5 icKO mice (Fig. 4A–4C), suggesting that *erk5* deletion actually increased, rather than decreased, the pool of proliferating cells in the SVZ. However, a smaller fraction of those Ki67^+^ cells in the ERK5 icKO mice were in the S-phase, manifested as a significant reduction in the number of BrdU and Ki67 co-labeled cells among total Ki67^+^ cells (Fig. 4D–4F). Consequently, ERK5 deletion did not change the output of actively proliferating, BrdU-incorporating S-phase cells (Fig. 4G–4I). These data suggest that although the cell cycle is likely altered upon *erk5* deletion, the reduced adult born neurons in the OB of ERK5 icKO mice is unlikely due to reduced proliferation in the SVZ.

### Deletion of *erk5* Inhibits Neuronal Differentiation and Maturation

**Figure 5:**
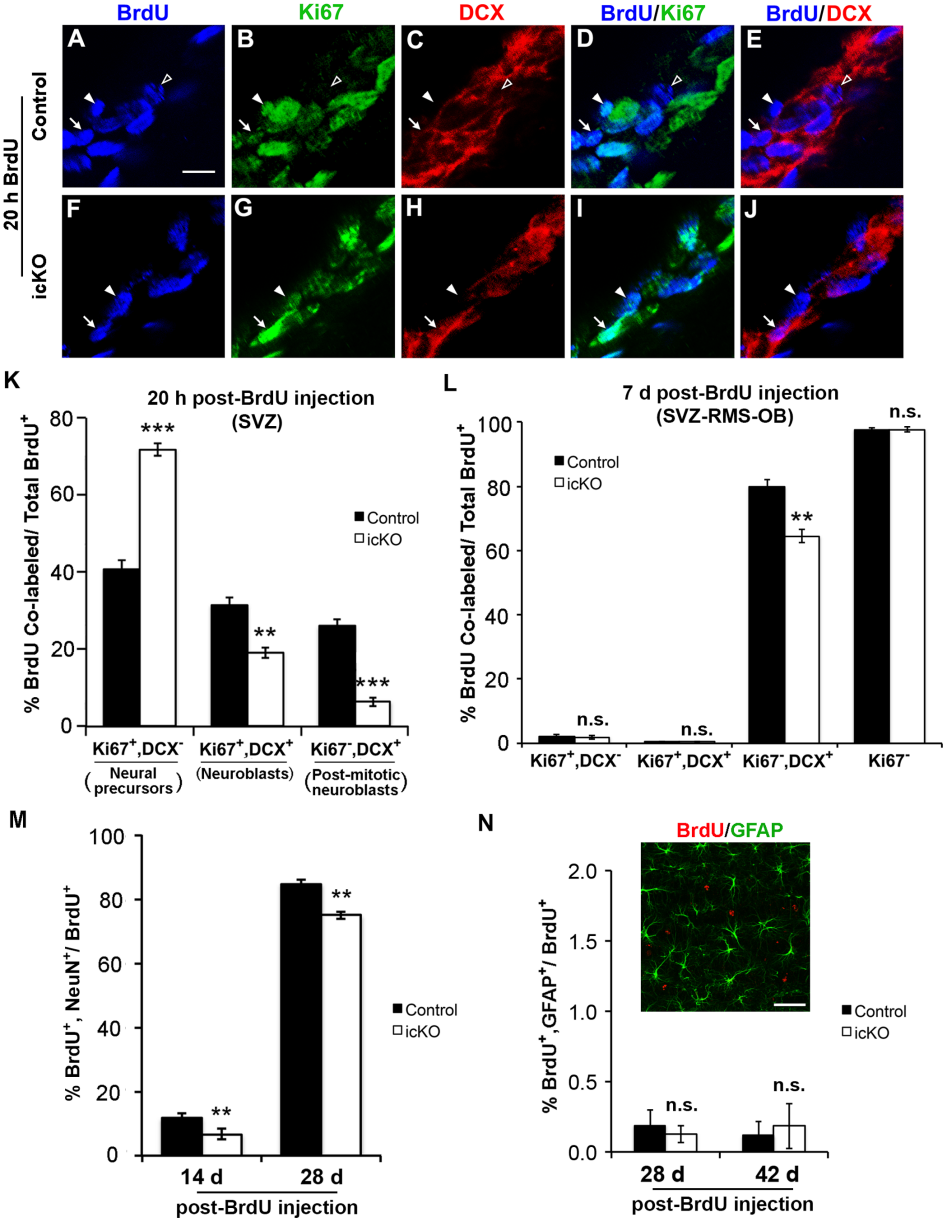
ERK5 deletion inhibits neuronal differentiation *in vivo* without affecting the production of astrocytes. (A–J) Representative confocal images of immunostaining for BrdU (pseudo-colored blue), Ki67 (green), and DCX (red) in the SVZ at 20 h post-BrdU injection. Filled arrowheads point to neural precursors (BrdU^+^, Ki67^+^, DCX^-^); arrows point to neuroblasts (BrdU^+^, Ki67^+^, DCX^+^); open arrowheads mark a post-mitotic neuroblast (BrdU^+^, Ki67^−^, DCX^+^). Scale bar represents 10 µm. (K) Quantification of the data from panels A-J as the percentages of neural precursors, neuroblasts, and post-mitotic neuroblasts in the BrdU^+^ population in the SVZ. (L) The percentage of BrdU^+^ cells that express Ki67 and/or DCX at 7 d post-BrdU injection along the SVZ-RMS-OB axis. (M) The percentage of BrdU^+^ population that express NeuN at 14 d or 28 d post-BrdU injection in the OB. (N) Representative confocal image of GFAP (green) and BrdU (red) co-staining in the OB at 28 d post-BrdU injection. Scale bar represents 40 µm. The graph shows the percentage of BrdU^+^ population that express GFAP at 28 d or 42 d post-BrdU injection in the OB. n  = 4 individual mouse brains and olfactory bulbs per group. n.s. not significant; **, p<0.01; ***, p<0.001.

The reduction of adult-born neurons in the OB of ERK5 icKO mice could result from decreased neuronal differentiation, survival, and/or from disruption of migration. Neuronal differentiation begins when neural precursors (BrdU^+^/Ki67^+^/DCX^-^) commit to a neuronal fate and become mitotic neuroblasts, manifested as expressing specific markers including DCX (BrdU^+^/Ki67^+^/DCX^+^). Neuroblasts can proliferate or exit the cell cycle, generating post-mitotic neuroblasts (Ki67^−/^DCX^+^), which then become immature neurons upon arrival at the OB. To examine a role for ERK5 in cell cycle exit and neuronal differentiation, mice were sacrificed 20 h after a single BrdU injection. Neuroblasts that have recently exited cell cycle to form post-mitotic neuroblasts would maintain their BrdU labeling but no longer express Ki67, thus newborn post-mitotic neuroblasts can be identified as BrdU^+^/Ki67^−/^DCX^+^. Of the total BrdU labeled cells, there was a 39% reduction of neuroblasts and a 76% increase in neural precursors in the SVZ of ERK5 icKO mice (Fig. 5A–5K), suggesting a delayed neuronal fate commitment of neural precursors to form neuroblasts. Furthermore, there was a 75% decrease in post-mitotic neuroblasts in ERK5 icKO mice, a rate much higher than the 39% decrease in mitotic neuroblasts. Thus, it is likely that *erk5* deletion delayed cell cycle exit of mitotic neuroblasts, thus inhibiting the formation of post-mitotic neuroblasts and consecutively the number of newborn neurons. The combined effect of inhibition of neuronal fate commitment of precursors and delayed cell cycle exit of mitotic neuroblasts likely contribute to inhibition of neuronal differentiation, and consequently, the reduction of newborn neurons in the OB of ERK5 icKO mice.

To further substantiate the finding of *erk5* deletion on neuronal differentiation, we quantified the number of immature and mature neurons at 7, 14, and 28 d post-BrdU injection. Almost all BrdU^+^ cells lacked Ki67 expression at 7 d post-BrdU labeling, and were thus post-mitotic (Fig. 5L). However, fewer BrdU-labeled, Ki67^−^ postmitotic cells expressed DCX in the SVZ-RMS-OB axis of ERK5 icKO mice (Fig. 5L), indicating a reduction of immature neurons. In addition, there was a decrease of BrdU-labeled cells that expressed NeuN at 14 d and 28 d post-BrdU injection (Fig. 5M).

To examine if *erk5* deletion alters glial differentiation, OB sections were co-stained for BrdU and GFAP, a marker for astrocytes in the OB. Very few (less than 0.2%) BrdU^+^ cells were co-labeled with GFAP in the OB of control mice at 28 d and 42 d post-BrdU injection (Fig. 5N), consistent with other studies [35], [36]. Deletion of *erk5* did not significantly change this number. Thus *erk5* deletion inhibits neuronal differentiation without affecting astroglial differentiation.

**Figure 6:**
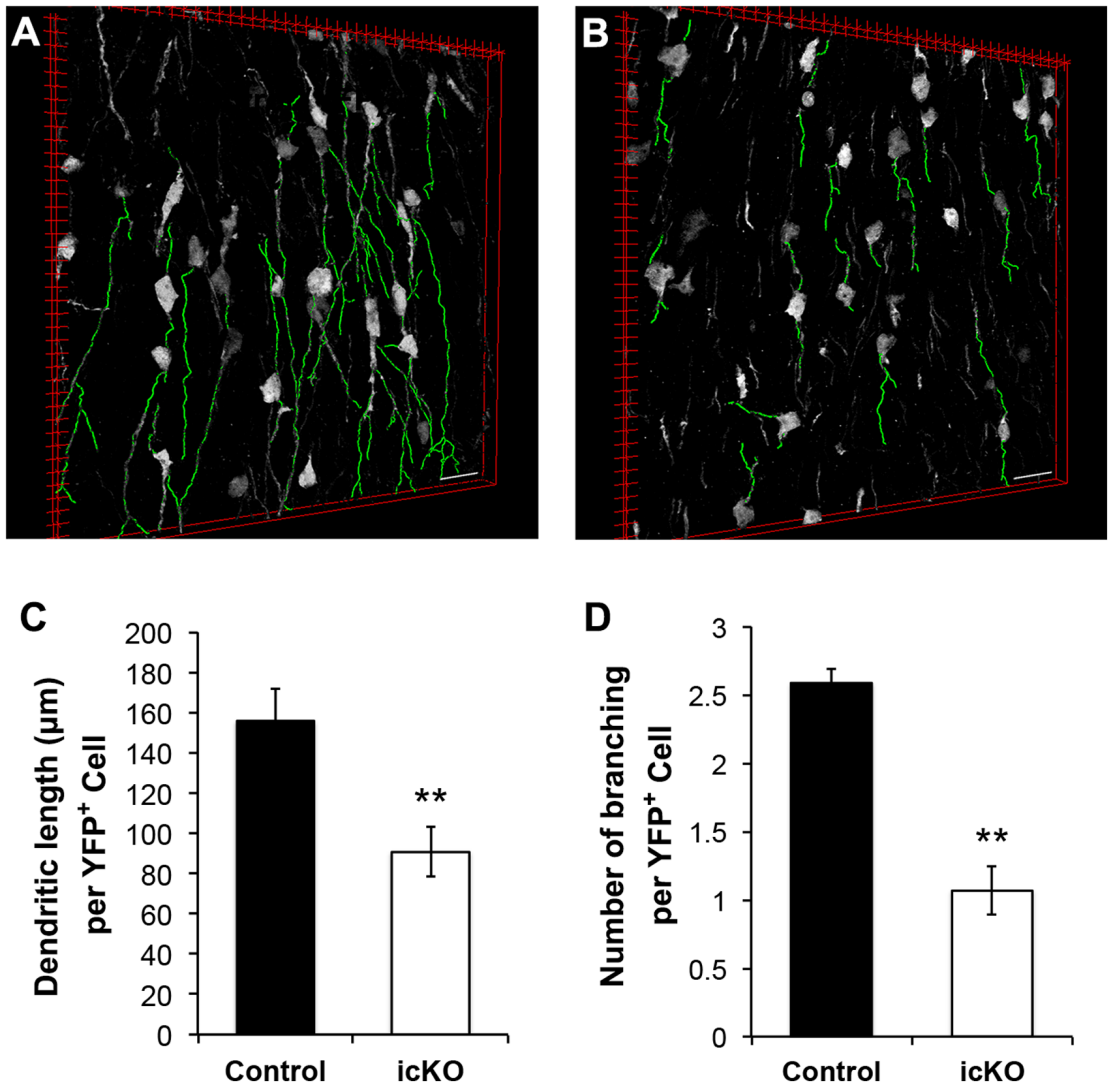
ERK5 deletion inhibits neuronal maturation in the OB. (A–B) Representative 3-D images of YFP^+^ cells in the OB. The OB sections were cut at 20 µm. Dendrites were highlighted by green color using Simple Neurite Tracer. Images were created by the 3D viewer of ImageJ. Scale bars represent 25 µm. (C) Average dendritic length of YFP^+^ cells in the OB was measured. (D) Quantification of the average number of dendritic branching of YFP^+^ cells. n  = 3 individual mouse brains and olfactory bulbs per group. **, p<0.01.

To address whether ERK5 regulates neuronal maturation once newborn neurons are in the OB, we crossed the Nestin-CreER™/ERK5^loxP/loxP^ mice into the R26-YFP^loxP/loxP^ background and obtained Nestin-CreER™/ERK5^loxP/loxP^/R26-YFP^loxP/loxP^ mice. Nestin-CreER™/R26-YFP^loxP/loxP^ littermates were used as controls. Both mice were treated with tamoxifen for 7 d to induce Cre-mediated recombination; animals were sacrificed 32 d after last dose of tamoxifen. The expression of YFP facilitates the detection of morphological changes in adult-born neurons that either contain *erk*5 in Nestin-CreER™/R26-YFP^loxP/loxP^ mice or are deleted of *erk*5 in Nestin-CreER™/ERK5^loxP/loxP^/R26-YFP^loxP/loxP^ mice. The dendritic length and branching number were quantified as a measure of neuronal maturation [16], [37]. Deletion of *erk*5 significantly decreased the dendritic length and the number of branching of adult-born neurons (Fig. 6). These data suggest a critical role for ERK5 in neuronal maturation.

### ERK5 Knockout Disrupts Both Chain and Radial Migration of Adult-born Cells

**Figure 7:**
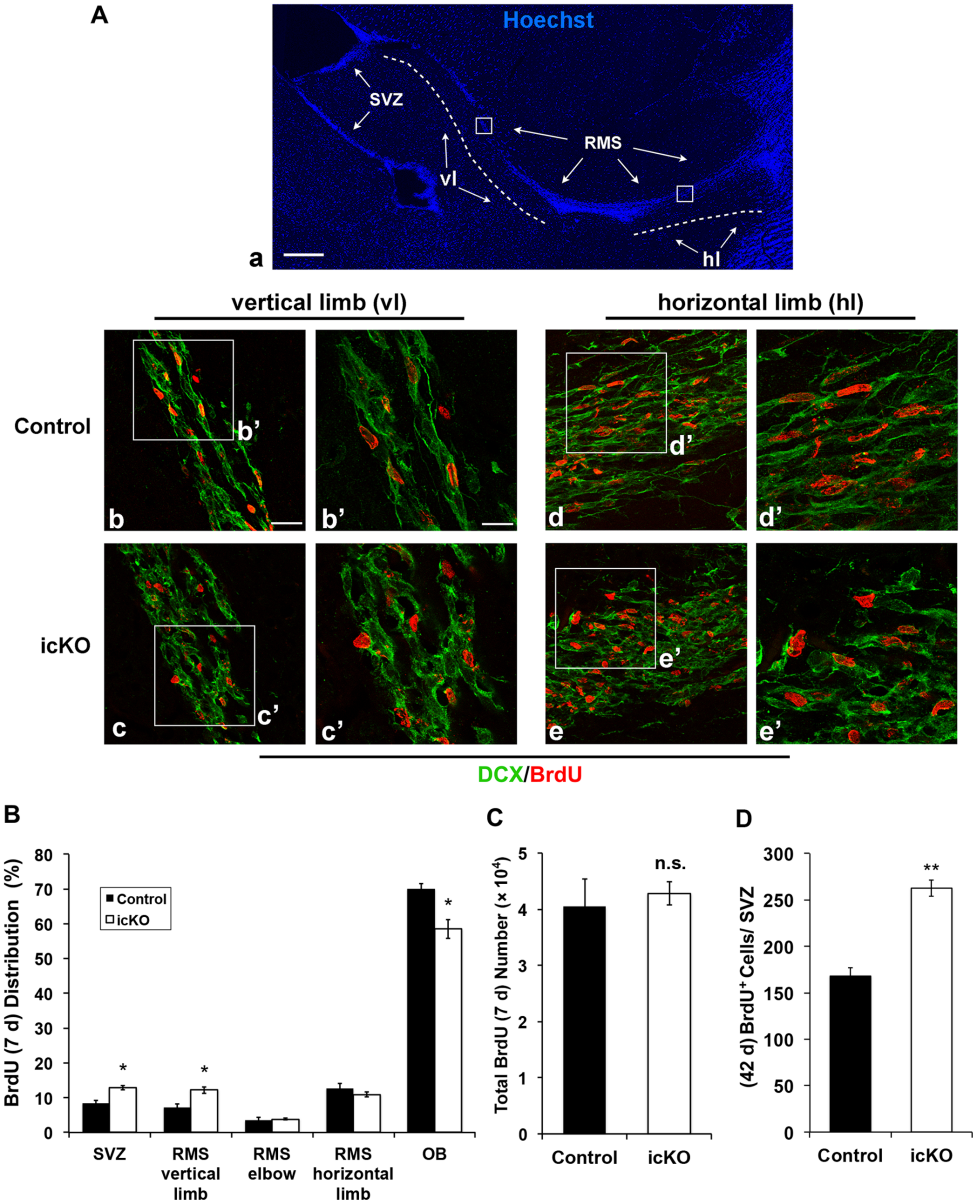
ERK5 icKO impairs chain migration of newborn cells in the RMS. (A) Immunohistochemistry staining of the RMS with DCX (green) and BrdU (red), 7 d post-BrdU injection. Hoechst staining (Blue) was used to visualize the SVZ-RMS-OB pathway in (a). Dash lines in (a) show the vertical limb (vl) and horizontal limb (hl) of the RMS. Scale bars in (a) represents 250 µm, in (b) represents 25 µm and applies to (b-e), in b’ represents 12.5 µm and applies to (b’-e’). Squares in (b-e) were enlarged in (b’-e’), respectively. (B) Quantification of regional BrdU^+^ cell distribution along the SVZ-RMS-OB pathway at 7 d post-BrdU injection. (C) Quantification of total BrdU^+^ cells along the SVZ-RMS-OB pathway at 7 d post-BrdU injection. (D) Quantification of BrdU^+^ cells in the SVZ at 42 d post-BrdU injection. n  = 4 individual mouse brains and olfactory bulbs per group. n.s. not significant; *, p<0.05; **, p<0.01.

Neuroblasts migrate tangentially (chain migration) along the RMS. Once reaching the core of the OB, they become immature neurons and switch their orientation and migrate radially to their final destinations in different layers of the OB. Since DCX is expressed in neuroblasts, we examined the morphology of DCX^+^ cells in the RMS. DCX^+^ cells of control mice, in general as well as those co-labeled with BrdU, were elongated with leading processes, and lined up orderly parallel to the RMS (Fig. 7A). In contrast, DCX^+^ cells in ERK5 icKO mice were disorganized, more rounded, and lacked leading processes. These morphological changes indicate altered migration [38]. These results suggest an impaired chain migration in the RMS of ERK5 icKO mice, which could result in less efficient migration and delay of adult-born cells reaching the OB, thus contributing to the reduction of adult-born neurons in the OB. Indeed, when we examined the relative distribution of adult-born cells at 7 d post-BrdU injection along the SVZ-RMS-OB axis, more BrdU^+^ cells remained in the SVZ and the vertical limb of the RMS, while fewer reached the OB in ERK5 icKO mice (Fig. 7B), even though the total number of BrdU^+^ cells was similar between control and ERK5 icKO mice (Fig. 7C). Moreover, there were still more BrdU^+^ cells in the SVZ even 42 d after BrdU injection (Fig. 7D).

**Figure 8:**
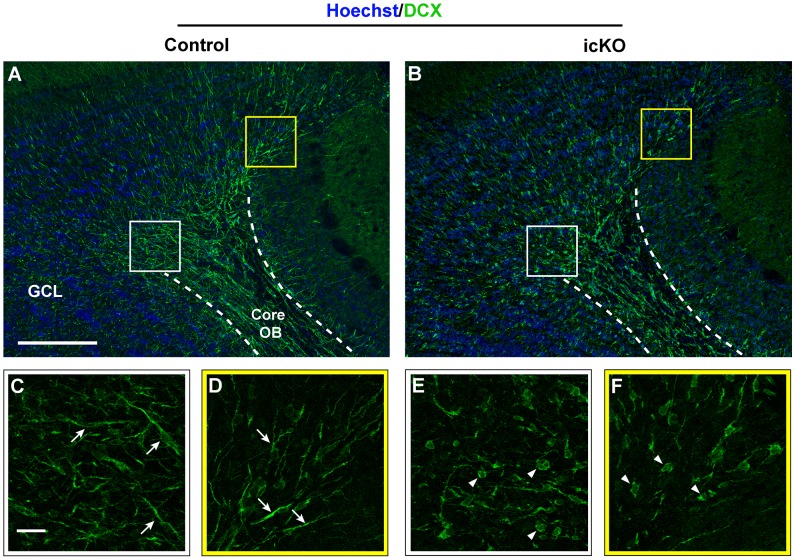
ERK5 icKO impairs radial migration of newborn cells in the OB. Sagittal sections from control and ERK5 icKO brains were stained for Hoechst (blue) and DCX (green), to examine the morphology of newborn cells radially migrating away from the core of the OB to the GCL. Scale bar in (A) represents 250 µm and applies to (B). White and yellow squares in (A, B) were closely analyzed by confocal stacks and shown in (C-F), respectively. Scale bar in (C) represents 25 µm and applies to (C-F). Arrows point to elongated DCX^+^ cells with leading processes. Arrowheads point to round DCX^+^ cells lacking leading processes.

Similarly, DCX^+^ cells that were undergoing radial migration out of the core of the OB lacked leading processes, had rounded cell bodies and were dis-arrayed in ERK5 icKO mice compared to those in control mice (Fig. 8). These data suggest that ERK5 deletion impairs both chain and radial migration.

### ERK5 Knockout Decreases Survival of Newborn Cells in the OB

**Figure 9:**
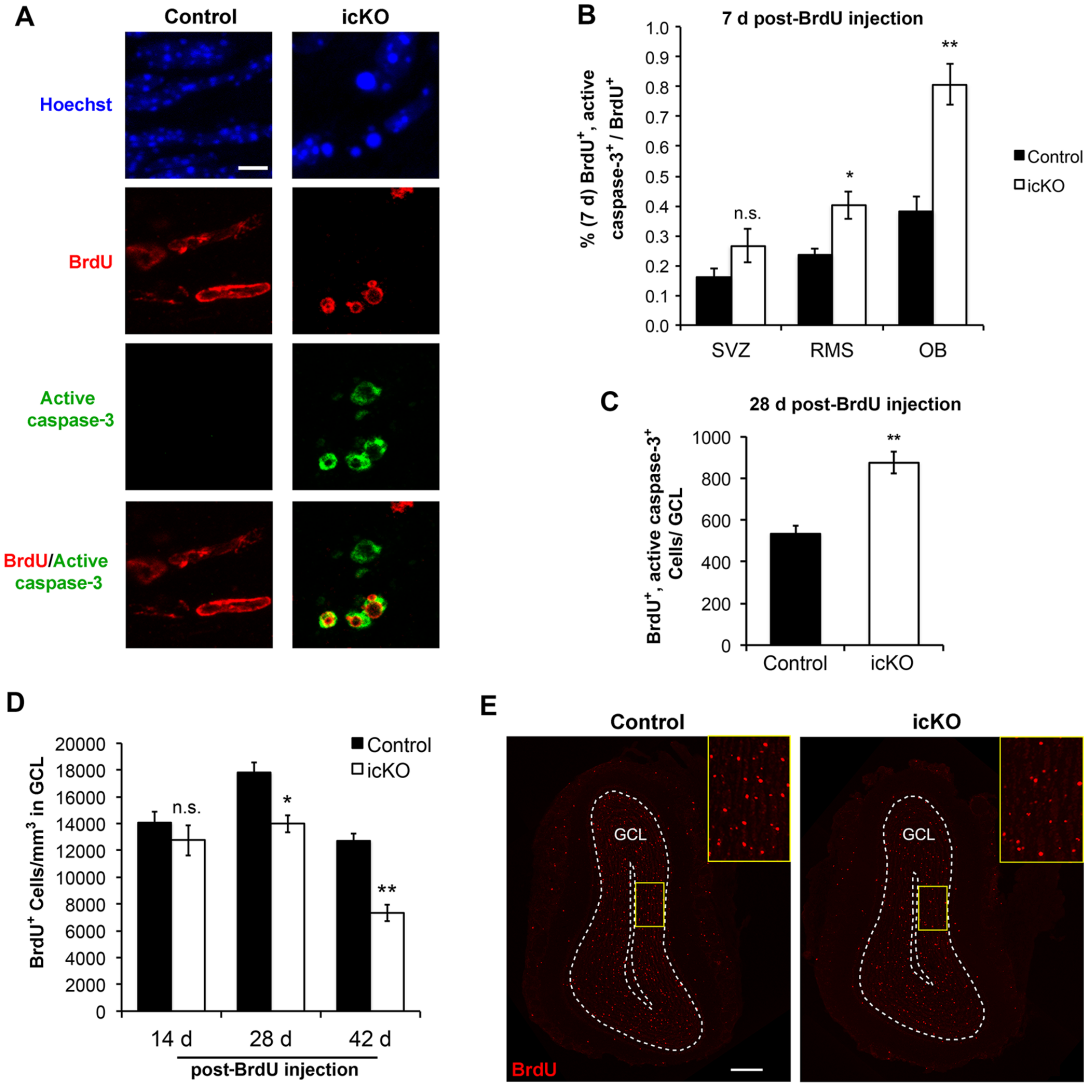
ERK5 icKO inhibits survival of newborn cells in the OB. (A) Representative immunostaining of active caspase-3 (green) and BrdU (red) at 7 d post-BrdU injection in the OB. Hoechst stained nuclei (blue). Scale bar represents 5 µm and applies to all panels. (B) Quantification of active caspase-3^+^ cells in BrdU^+^ cell population at 7 d post-BrdU injection. (C) Quantification of total active caspase-3^+^ and BrdU^+^ cells at 28 d post-BrdU injection. (D) Quantification of BrdU^+^ cell density in the GCL of the OB, at 14, 28 and 42 d post-BrdU injection. (E) Representative images of BrdU staining (red) at 28 d post-BrdU injection. The yellow rectangles outline the enlarged areas, shown in the insets, for clearer visualization. Scale bar represents 250 µm. n  = 4 individual olfactory bulbs per group. n.s. not significant; *, p<0.05; **, p<0.01.

We next used active caspase-3, a marker for apoptosis, and BrdU co-staining to identify adult-born, apoptotic cells. Targeted deletion of *erk5* increased the percentage of adult-born cells undergoing apoptosis in both the RMS and OB 7 d post-BrdU injection (Fig. 9A–9B). To examine cell survival over a longer timeframe, we quantified the number of BrdU^+^ and active caspase3^+^ cells in the OB 28 d post-BrdU injection, as well as the density of BrdU^+^ cells in the GCL of OB at 14 d, 28 d and 42 d post-injection. There were 58% more BrdU^+^ and active caspase-3^+^ cells in the OB of ERK5 icKO mice 28 d post-BrdU injection (Fig. 9C). Although the density of BrdU^+^ cells was comparable at 14 d in control and ERK5 icKO mice, it was significantly lower in ERK5 icKO OB at 28 d and 42 d (Fig. 9D–9E).

**Figure 10:**
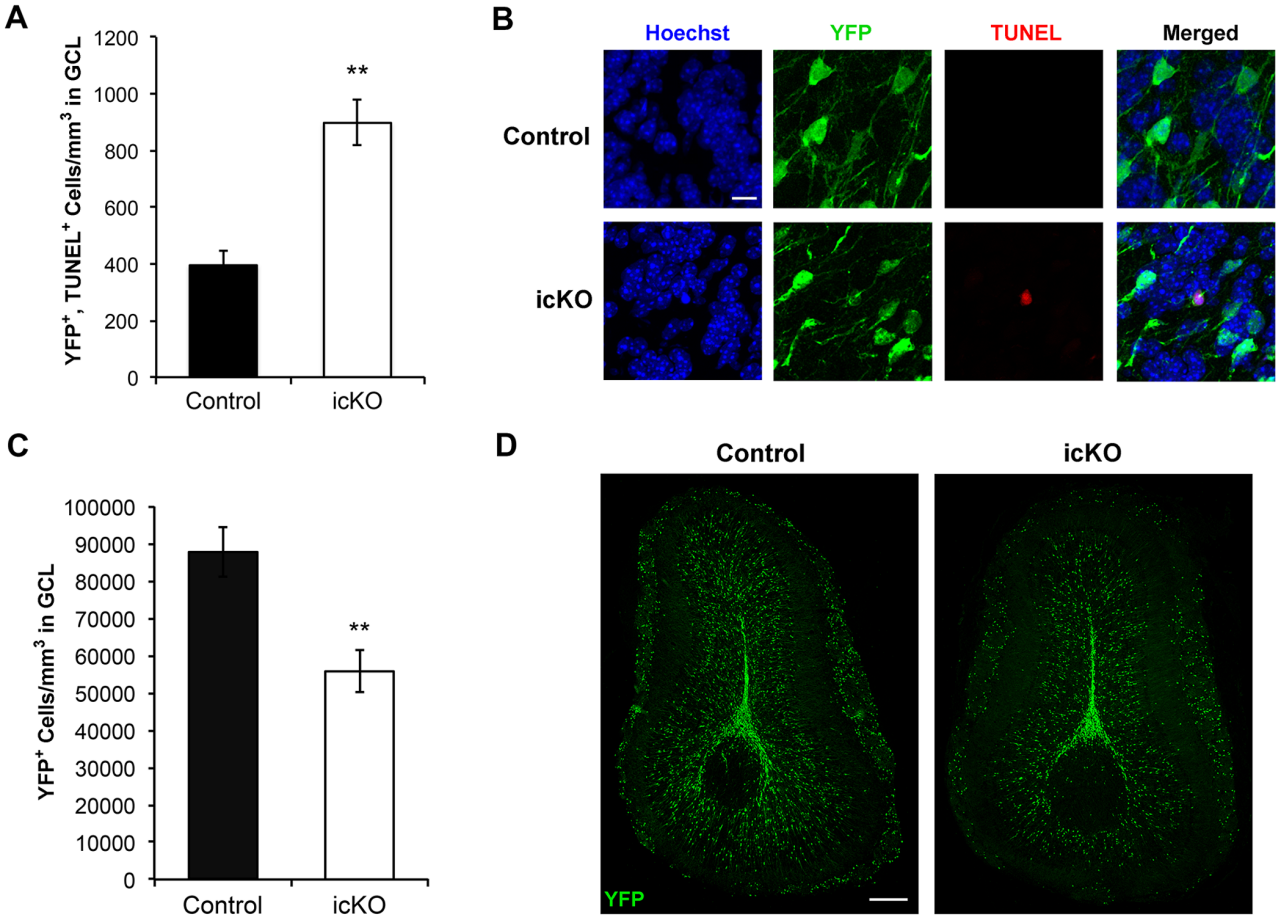
ERK5 icKO inhibits survival of newborn cells in the OB of YFP reporter mice. (A) Quantification of the density of YFP^+^ cells in the GCL of control (Nestin-CreER™/R26-YFP^loxP/loxP^) and ERK5 icKO mice (Nestin-CreER™/ERK5^loxP/loxP^/R26-YFP^loxP/loxP^) that are also TUNEL^+^, at 32 d after the last dose of tamoxifen. (B) Representative images of Hoechst (blue), YFP (green), and TUNEL (red) staining in the GCL. Scale bar represents 10 µm and applies to all images. (C) Quantification of YFP^+^ cell density in the GCL at 32 d after the last dose of tamoxifen. (D) Representative images of YFP immunostaining in the OB. Scale bar represents 250 µm and applies to both images. n  = 3 individual olfactory bulbs per group. **, p<0.01.

To further confirm the findings of *erk5* deletion on increased neuronal death, we used the YFP-reporter mice to examine the survival of newborn cells in the OB. ERK5 knockout significantly increased the density of YFP^+^ cells in the GCL of the OB that were positive for TUNEL (Fig. 10A–10B), another measure of apoptosis. The density of YFP^+^ cells in the GCL of the OB, 32 d after last tamoxifen dosing, was lower in ERK5 icKO mice (Nestin-CreER™/ERK5^loxP/loxP^/R26-YFP^loxP/loxP^) than in control littermates (Nestin-CreER™/R26-YFP^loxP/loxP^) (Fig. 10C–10D). Together, these data implicate a critical role for ERK5 in cell survival during adult neurogenesis in the SVZ-RMS-OB axis.

## Discussion

In rodents, the periglomerular cells and granule cells of the OB, both interneurons, are continuously generated throughout life. Newly generated neurons in the adult OB have been implicated in OB function and some complex forms of olfactory behavior [11], [12], [13], [14], [15]. Although adult-born neurons in the OB have been extensively characterized at the cellular level, signaling mechanisms regulating adult OB neurogenesis are not well defined. We discovered that although the ERK5 MAP kinase is highly expressed throughout the entire brain during development, its expression in the adult brain is restricted to adult neurogenic regions including the SVZ [25]. This striking and unique pattern of expression suggests a fundamentally important role for ERK5 in neurogenesis. The objective of this study was to investigate the role of ERK5 in the regulation of adult OB neurogenesis, and specifically, to examine which aspects of adult OB neurogenesis are regulated by ERK5 MAP kinase.

Using RNAi technology to suppress ERK5 expression or retroviral expression of caMEK5 to activate endogenous ERK5 signaling, we demonstrate that ERK5 is both necessary and sufficient for SVZ-derived aNPCs to differentiate into neurons in culture. Using transgenic mouse technology, we conditionally deleted the *erk5* gene in nestin-expressing neural stem/progenitor cells in the adult brain upon tamoxifen administration. Deletion of *erk5* reduced adult neurogenesis in the OB *in vivo*. These *in vitro* and *in vivo* data suggest a critical role for ERK5 in the regulation of adult OB neurogenesis.

Several growth factors and signaling molecules have been implicated in the proliferation of aNPCs in the SVZ and RMS, including sonic hedgehog (Shh), and Wnt/β-catenin/APC [5], [9], [39], [40], [41]. In non-neuronal cells, dominant negative suppression of ERK5 inhibits DNA synthesis and cell proliferation [34], [42]. Deletion of ERK5 from embryonic mouse brain also inhibits BrdU incorporation and cell proliferation in the SVZ and RMS [43]. Here we show that ERK5 is expressed in proliferating cells in the neurogenic SVZ-RMS of the adult brain. However, unlike in non-neuronal cells or in the developing brain, suppression of ERK5 signaling by gene deletion in adult neurogenic regions *in vivo* did not decrease the number of BrdU^+^ cells 2 h post-labeling, suggesting that *erk5* deletion does not inhibit proliferation. This idea is further supported by the fact that there is no difference in the density of BrdU^+^ cells in the OB 14 d post-labeling. Thus, the reduced OB adult neurogenesis of ERK5 icKO mice is not because of reduced proliferation in the SVZ.

We have previously implicated ERK5 in specifying cortical stem/progenitor cells toward a neuronal lineage, and in the differentiation of GABAergic interneurons in the OB during embryonic development [32], [43], [44]. Here we provide evidence that targeted deletion of *erk5* in adult neurogenic regions delays neuronal fate commitment of precursors to neuroblasts and cell cycle exit of neuroblasts, reduces the production of DCX-expressing immature neurons, NeuN-expressing mature neurons, and dendritic length and branching once newborn neurons reached the OB. Significantly, *erk5* deletion did not affect newborn GFAP^+^ glial cells in the OB. Together, these data suggest that *erk5* deletion specifically inhibits neuronal lineage commitment, neuronal differentiation, and neuronal maturation without affecting the production of astroglia in the adult OB. The fact that ERK5 also regulates neuronal differentiation in the SGZ [26] suggests a general role for ERK5 in promoting neuronal differentiation during developmental and adult neurogenesis.

Except for a few molecules, including ErbB4, Nogo A and Nogo receptor 1, plexin-b2, and MARK2 [45], [46], [47], [48], little is known about signaling mechanisms regulating cell migration during adult OB neurogenesis, especially the switch from chain to radial migration. Interestingly, DCX-expressing neuroblasts in the RMS and immature neurons in the OB of ERK5 icKO mice have altered organization, cell orientation, cell shape, and lack leading processes. Since similar morphological changes have been used as evidence for altered migration [38], these data implicate ERK5 in both chain and radial migration.

There were fewer 7-day old BrdU^+^ cells in the OB and more in the SVZ and RMS of ERK5 icKO mice. This change of relative distribution of BrdU^+^ cells further substantiates the conclusion that *erk5* deletion alters tangential migration. There was no net increase of BrdU^+^, actively proliferating cells in the SVZ (Fig. 4I), and at 7 d post-BrdU injection, almost all BrdU^+^ cells were post-mitotic and Ki67^−^ (Fig. 5L). Thus, it is unlikely that the increase of BrdU^+^ cells in the SVZ and RMS at 7 d post-BrdU injection is due to increased proliferation. Although there was an increase of apoptosis in the RMS and OB of ERK5 icKO mice 7 d post-BrdU injection, only a very small percentage of total BrdU-labeled cells were undergoing apoptosis at this time point, 0.4% and 0.8% for RMS and OB of ERK5 icKO mice, respectively. When compared to the control, there was only a 0.15% increase of apoptosis in the RMS and a 0.4% increase in the OB. This level of increase of apoptosis is too small to account for the 10% decrease of total BrdU^+^ cells in the OB (Fig. 7B). Furthermore, there was no change in the total number of BrdU^+^ cells 7 d post-BrdU injection (Fig. 7C). Together with the fact that there was no net increase of proliferation, these data further suggest that increased cell death alone was unlikely responsible for the 10% decrease of total BrdU^+^ cells in the OB. The more likely explanation is that *erk5* deletion caused a defect in tangential migration. Consequently, more newborn cells remained in the SVZ and RMS, fewer reached OB. In fact, there were more BrdU^+^ cells in the SVZ even 42 d after BrdU injection.

Many of the adult-born OB neurons die by apoptosis in an activity-dependent manner [16], [17]. One of the first signaling mechanisms implicated in the survival of adult-born OB neurons was the activation of the ERK1/2 MAP kinase after odor exposure [49]. The transcription factor CREB, whose activity is regulated by ERK1/2, also contributes to neuronal activity-dependent differentiation [50] and survival of adult-born neurons in the SVZ/OB [49], [50], [51], [52]. Consistent with its role in promoting the survival of several different types of newborn neurons including those in the OB during embryonic development [43], [53], [54], [55], [56], targeted deletion of *erk5* in adult neurogenic regions increased apoptosis of adult-born cells in the RMS and OB.

Adult neurogenesis is a dynamic process governed by various intrinsic and extrinsic factors. Previous studies have substantially enriched our understanding of intrinsic factors, many of which are transcription factors, which regulate adult OB neurogenesis. For example, transcription factors SOX2 and Tlx regulate the proliferation of neural precursors [57], [58], while Pax6, Dlx2, Tbr2 and CREB promote neuronal fate specification [51], [59], [60], [61], [62]. Moreover, Pax6 and CREB are also important for the survival of newborn neurons in the OB [49], [50], [51], [52], [63]. In addition to these transcription factors, a few signaling molecules have been implicated in adult OB neurogenesis. For example, Wnt and Notch regulate proliferation [64], [65]; Shh and Nogo have been implicated in the proliferation in the SVZ and migration in the RMS [66], [67], [68], and ERK1/2 in neuronal survival [49]. Nevertheless, much more research is needed to better define signaling pathways regulating adult OB neurogenesis.

ERK5 is a MAP kinase signaling pathway that is activated by a variety of receptors including those for growth factors, neurotrophic factors, cytokines, and G-protein coupled receptors [69], [70], [71]. We recently demonstrated that ERK5 is activated by prolactin in the SVZ, which underlies prolactin- and mating-induced adult neurogenesis in the OB [27]. These findings identify ERK5 MAP kinase as a strong candidate for regulation of adult OB neurogenesis in response to extracellular signals. The specific pattern of expression in adult neurogenic regions also distinguishes ERK5 from other signaling molecules implicated in adult SVZ-OB neurogenesis, such as BMP, Shh, PTEN, and Wnt, which are more widely expressed in the brain [66], [72], [73], [74], [75], [76], [77], [78], [79], [80], [81], [82], [83]. Another unique feature of ERK5 signaling is its role in regulating multiple aspects of adult OB neurogenesis including neuronal commitment and differentiation, migration, and survival. Finally, ERK5-regulated adult OB neurogenesis has a documented role in olfactory behavior [15].

Data presented here demonstrated that *erk5* deletion decreases OB adult neurogenesis through mechanisms including inhibition of neuronal fate commitment, neuronal differentiation and maturation, neuronal survival, and impaired migration both in the RMS and in the OB. Although cell cycle was altered in the SVZ of ERK5 icKO mice, reduced OB neurogenesis cannot be attributed to reduced proliferation because proliferation was not reduced. It is technically infeasible to eliminate the possibility that the observed effect on each and any of the different stages of OB neurogenesis is not the simple consequence of the effect of ERK5 on an earlier stage. For example, ERK5 is expressed in neuroblasts that are proliferative and migrate along RMS. These neuroblasts also undergo neuronal differentiation along the way. For instance, the Type A migratory neuroblasts in the RMS are mitotic, migratory, and express DCX, PSA-NCAM and calretinin. It would be impossible to selectively inhibit the function of ERK5 in one of the processes, such as proliferation, without affecting the other processes such as migration in the same cell. Nevertheless, it seems unlikely that the observed effect on newborn neuron survival is the consequence of the effect of ERK5 on cell proliferation. Increased apoptosis was seen in ERK5 icKO mice 7 d post-BrdU injection, a time point when almost all BrdU^+^ cells have exited cell cycle and are Ki67^−^ (Fig. 5L). There is also increased apoptosis of newborn cells in the OB of ERK5 icKO mice at 28 d post-BrdU injection (Fig. 9 C), a time point long after all newborn neurons have exited cell cycle. Although these data do not rule out the possibility that altered cell cycle regulation could affect cell survival of mitotic or newly postmitotic neuroblasts, it seems likely that *erk5* deletion adversely decreases the survival of newborn neurons independently of cell proliferation. Regardless, it is clear that targeted deletion of *erk5* led to an impairment in neuronal differentiation, migration, and survival during adult OB neurogenesis.

We did not observe any decrease of survival of newborn cells between 14 and 28 days after BrdU injection in control mice. This result may seem to contradict a report that showed a 25% reduction of BrdU-labeled cells during this period of time [19]. However, 8-week-old, C57BL/6 mice were used in the previous report [19]. Our ERK5 icKO mice are in a mixed SV129/C57BL/6 genetic background. To delete *erk5*, tamoxifen was administered to 10–12-week-old mice once a day for 4 days, repeated for 3 rounds with a 2-week recovery period between each round of tamoxifen dosing. BrdU was injected 4 days after the last dose of tamoxifen, at which time mice were 16–18-week-old. The differences in the genetic background and the age of the mice may explain the seemly different results reported in our study and that in the literature. Indeed, Yamaguchi et al reported that at day 14 post-BrdU injection, about 80% of BrdU^+^ cells were already NeuN^+^ [19]. However, only 12% of BrdU^+^ cells expressed NeuN at day 14 under our experimental conditions; it was not until day 28 post-BrdU injection that about 80% of the BrdU-labeled cells were NeuN^+^ in our mice (Fig. 5M). Interestingly, Yamaguchi observed a 25% decrease of the total number of BrdU^+^ cells from day 14 to day 28 post-BrdU injection. We report a 28% decrease of the total number of BrdU^+^ cells from day 28 to day 42 post-BrdU injection in control mice (Fig. 9D). Thus, once 80% of the adult-born cells are NeuN^+^–day 14 in Yamaguchi’s study or day 28 in this report, 25%–28% of the adult-born cells die within a 2-week period. This conclusion indicates that data reported here are in fact consistent with existing literature [19] and with the idea that newborn neurons undergo apoptosis in an activity-dependent manner [16], [17]. Our data suggest that aging may slow down the rate of neuronal differentiation of adult born neurons. This notion is consistent with the report that aging decreases adult neurogenesis by events downstream of proliferation [84].

ERK5 regulates proliferation, neuronal differentiation, and survival during embryonic development [32], [43], [44], [53], [54], [55], [56], and neuronal differentiation in adult SGZ [26]. Together with data presented here, our findings suggest a critical role for ERK5 in the regulation of neurogenesis both during embryonic development and in the adult brain, and support the notion that developmental and adult neurogenesis share some common signaling mechanisms including ERK5 signaling. It will be interesting in the future to investigate activators and downstream targets of ERK5 signaling that control the multiple steps of adult neurogenesis.
